# Prehospital emergency nurses’ experiences of caring for patients with suspected acute myocardial infarction: an interview study

**DOI:** 10.1136/bmjopen-2024-088754

**Published:** 2024-09-10

**Authors:** Sebastian Bjöhle, Veronica Vicente, Caroline Eriksson, Katarina Bohm, Maja Dodd, Rebecka R. Wahlin, Jakob Lederman

**Affiliations:** 1 Department of Clinical Science and Education, Södersjukhuset, Karolinska Institute, Stockholm, Sweden; 2 AISAB Ambulance Care in Greater Stockholm Ltd, Stockholm, Sweden; 3 Department of Medicine, Karolinska Institute, Huddinge, Sweden

**Keywords:** accident & emergency medicine, nurses, myocardial infarction, qualitative research, cardiology

## Abstract

**Abstract:**

**Objective:**

Despite the prevalence of emergency medical service assignments related to chest discomfort, limited research delves into nurses’ experiences of caring for such patients. This study aimed to illuminate prehospital emergency nurses’ (PENs’) experiences of caring for patients with suspected acute myocardial infarction (AMI).

**Design:**

A qualitative interview study.

**Setting:**

Two Swedish emergency medical service organisations in two different regions.

**Participants:**

Consecutive inclusion of 12 PENs.

**Data analysis:**

An inductive content analysis according to Elo and Kyngäs.

**Results:**

The results underline the active role of PENs in providing care for patients with AMI in the emergency medical service. They emphasise the ability to identify classic symptoms and the need for an open-minded approach to diffuse presentations. Ensuring patient security, projecting knowledge and composure are decisive for instilling a sense of safety. Healthcare providers feel profound responsibility and a fear of errors, especially in critical situations with potential patient deterioration. Prioritisation in time-sensitive cases leans towards medical interventions and immediate transportation. Continuing education is essential to enhance patient management and safety. Effective communication and trust are vital for urgent patient care, and prompt activation of the ST-elevation myocardial infarction pathway is recognised as imperative. Malfunctions result in frustration, underlining the importance of pathway functionality.

**Conclusions:**

PENs have immense responsibility for the swift and knowledgeable management of patients with suspected AMI. Balancing patient involvement and urgent medical measures is challenging, emphasising the critical role of mental preparedness and comprehensive education. The study underlines the significance of effective communication and trust between healthcare providers, particularly in time-sensitive scenarios. Establishing feedback mechanisms for prehospital staff is important for advancing prehospital emergency care in this patient category.

**STRENGTHS AND LIMITATIONS OF THIS STUDY**

- The open character of this interview study allowed our participants to reflect on and deepen their own understanding of their experiences of caring for patients with acute myocardial infarction, resulting in rich and variated descriptions.
- Interviews were conducted solely with prehospital emergency nurses (PENs), who are medically responsible for the decisions made by the EMS team.
- A diverse sample was ensured by recruiting participants from different age groups, genders and working experience levels from two distinct regions.
- One limitation could be the small sample size of 12 participants; however, the enriching nature of the content is ultimately more significant.
- The study’s data was collected before COVID-19, and although changes in patient care-seeking behaviour have been documented, potential alterations in PENs’ experiences during the pandemic remain unexplored.

## Introduction

 Acute myocardial infarction (AMI) demands immediate attention. In Sweden, around three people experience an AMI every hour,^1^ with many not surviving until hospital arrival. It is therefore crucial for the emergency medical services (EMS), typically the initial medical contact, to identify and attend to these patients efficiently, impacting individuals of various age groups, including younger and middle-aged individuals.^2^

The primary symptom is acute chest discomfort, often characterised as pain, pressure or tightness.^3 4^ Patients with these symptoms are advised to contact the EMS through the national emergency number 112 for prompt assessment and treatment of their suspected AMI.^5^ Given that 10% of all annual EMS assignments involve chest discomfort, this constitutes the largest patient group in the EMS.^6^ Sweden employs a nurse-based EMS system, with the highest formal competency being the prehospital emergency nurse (PEN). This registered nurse (RN) holds a Bachelor of Science Degree in Caring Science/Nursing and has completed a specialist ambulance nurse education, totalling an extra 60 credits.^7^ The PEN assumes ultimate responsibility for EMS team care, integrating both medical and nursing knowledge to assess patients of all ages, with diverse conditions, and in various settings.^8 9^

When encountering patients with acute chest discomfort, the PEN currently has limited tools for assessment, primarily relying on ECG registration and interpretation to identify patients with acute coronary syndrome, including AMI.^5 10^ According to the established ST-elevation myocardial infarction (STEMI) pathway, for cases in which the patient exhibits chest pain or the PEN suspects AMI, or when there are ST-elevations present, the ECG is transmitted to the hospital for interpretation by a cardiologist. The decision to activate the STEMI pathway is made by the cardiologist, primarily based on the ECG findings. The prompt initiation of the STEMI pathway not only reduces treatment delays but also reduces patient mortality. However, the majority of patients with AMI do not present with ST-elevation meeting STEMI criteria, rendering them ineligible for the established STEMI pathway. Thus, most patients with suspected AMI lack specific ECG findings, necessitating evaluation at the emergency department (ED), regardless of discharge diagnosis.^5^

Despite the high prevalence of chest discomfort-related EMS assignments, few studies describe nurses’ experiences of caring for such patients.^11^ This study is therefore significant as it complements existing research and offers new insights into the intricate nature of caring for patients with suspected AMI. By illuminating the experiences of PENs in the EMS, we hope to improve and highlight the understanding of caring for these patients. The results of this study could also explain findings from previous and future quantitative studies.

## Method

### Design and setting

This qualitative descriptive study employed an inductive approach to gain an in-depth understanding^12^ of PENs’ experiences of caring for patients with suspected AMI, due to the lack of prior knowledge in this underexplored area. The study adhered to the criteria for reporting qualitative research.^13^ The research took place in two distinct regions in Sweden, namely Region Stockholm (urban) and Region Dalarna (rural), where the former necessitates a minimum of one PEN, and the latter mandates a presence of an RN in each ambulance.

### Patient and public involvement

No patients were involved in this study.

### Sample

Recruitment occurred through local advertisements at the ambulance departments in both regions, with potential participants reaching out to the authors. Strategic sampling was employed for inclusion, requiring participants to possess a minimum of 1 year of working experience as a PEN. A total of 12 participants aged 29–53 years were included, with varying EMS work experience ranging from 2 to 14 years, see table 1.

**Table 1:** Participants’ characteristics

	Total (n=12)	Female (n=8)	Male (n=4)
Age (median, years)	34	34	34
Years in EMS (median)	6	6	6
Region Stockholm (n)	8	6	2
Region Dalarna (n)	4	2	2

EMSemergency medical services

### Data collection

Data collection took place face to face at the participants’ respective workplaces between 3 and 19 April 2019 most often before or after their work shift. The interviews were conducted by the main author SB and CE after being trained in interview technique by VV and KB. Both SB and CE were RNs enrolled in education to become PENs when interviews were conducted. A semistructured interview approach with an interview guide (see online supplemental appendix 1) was used, starting with the study aim and a context reminder. The interview guide was tested in a pilot interview, an interview that was also included in the analysis. Throughout the process, we examined and questioned our prior understanding and judgments during research team meetings and discussions to support reflexivity and trustworthiness of the data collection and analysis. This was achieved through research team discussions, balancing subject knowledge and openness. Saturation^14^ was reached after 10 interviews, with 2 additional interviews conducted to confirm saturation. This was validated in research team discussions. The interviews, lasting between 15 and 60 min (mean 27.5 min), were voice recorded and transcribed verbatim. Only the participant, SB and/or CE was present during the interviews.

### Data analysis

Content analysis, using an inductive approach per Elo and Kyngäs,^12^ comprised three phases: preparation, organisation and reporting. In the preparation phase, interviews were transcribed and thoroughly read through for content understanding by three authors (SB, CE and VV). In the organisation phase, meaning units aligning with the study aim were identified and coded. Codes were categorised into seven subcategories based on differences and similarities, with independent analysis adjusted until consensus was reached. In the reporting phase, content essence was categorised into generic and main categories. Participant quotations were provided with participants IDs and included for explicit examples.

## Results

**Figure 1:**
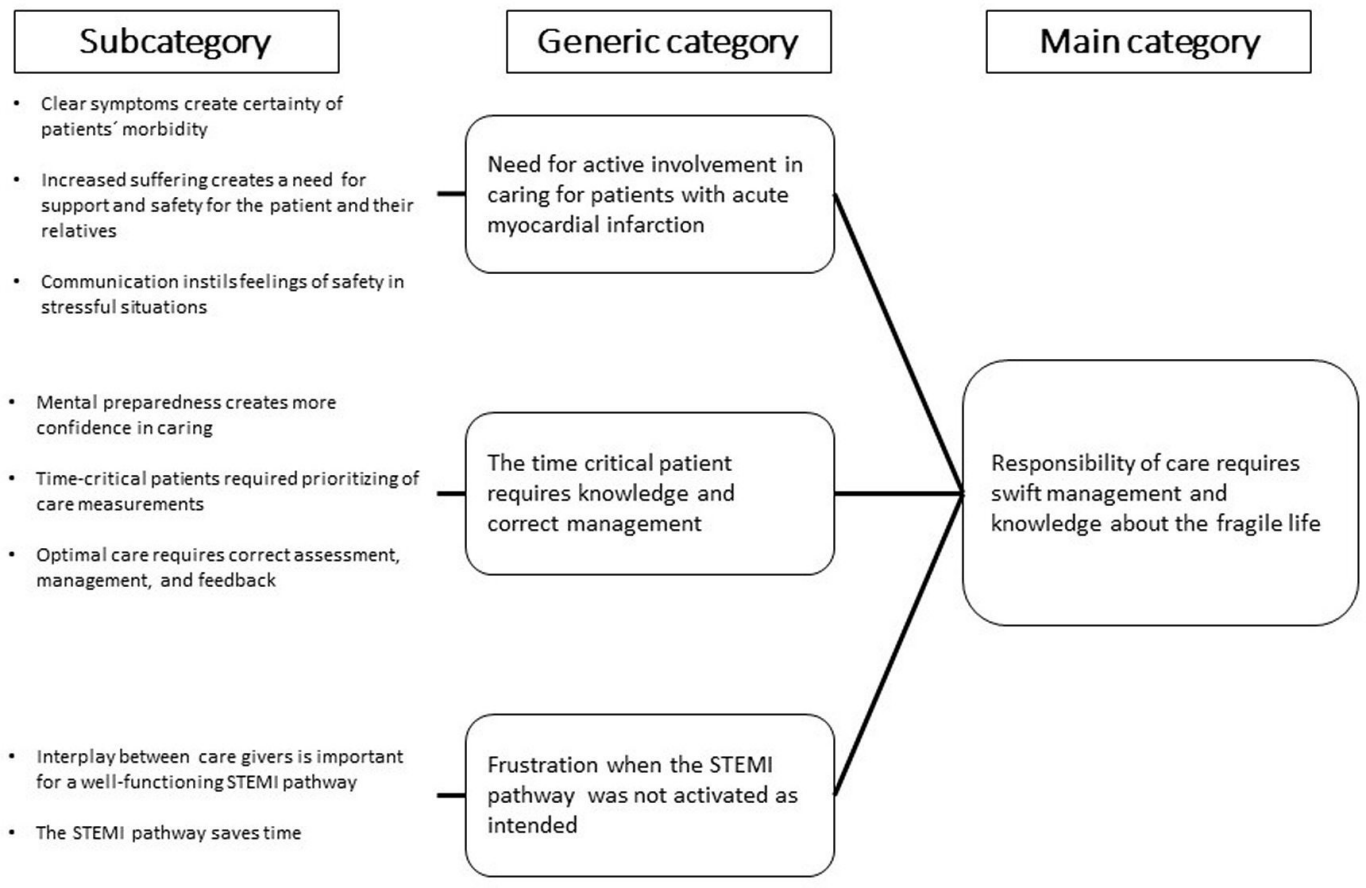
Subcategories, generic categories describing the main category based on prehospital emergency nurses’ experiences of caring for patients with suspected acute myocardial infarction. STEMI, ST-elevation myocardial infarction.

The essence of the experience of caring for patients with AMI in the EMS was described in the main category ‘Responsibility of care requires swift management and knowledge about the fragile life’. The three generic categories that underpin the main category were identified as: ‘Need for active involvement in caring for patients with AMI’, ‘The time-critical patient requires knowledge and correct management’ and ‘Frustration when the STEMI pathway was not activated as intended’. These three generic categories are presented below with the associated subcategories illustrated by quotations, see figure 1.

### Need for active involvement in caring for patients with AMI

This generic category underlines active involvement in providing care for patients with AMI in the EMS setting. It emphasises that patients with classic symptoms can often be identified from a distance. However, a more diffuse presentation of symptoms requires participants to adopt an open-minded approach in their assessment. Furthermore, it also stresses the importance of ensuring that the patient feels secure. Otherwise, non-compliance may pose challenges in care. Additionally, being perceived as knowledgeable and composed is essential to instil a sense of safety and involvement in both the patient and their relatives. The generic category is delineated by the subcategories: ‘Clear symptoms create certainty of patients’ morbidity’ and ‘Increased suffering creates a need for support and safety for the patient and their relatives’.

#### Clear symptoms create certainty of patients’ morbidity

The participants felt increased confidence in assessing the patient’s condition when presented with classical symptoms. Knowledge of classic myocardial infarction symptoms was notably high in the patients encountered in the care setting. At the onset of the care encounter, participants observed that patients expressed fear of experiencing an AMI. The participants experienced that patients with obvious AMI showed classical symptoms of AMI such as paleness, cold sweatiness, anxiety, nausea and a collapsed body posture. The classical pain radiating to the left arm also led to the rapid identification of the suspected AMI. One participant stated:

I think the obvious heart attacks are easy to identify if you have seen enough, you know somewhere that the symptoms are pointing to heart attack. You see they have cold sweat, are pale and anxious and perhaps nauseous and that. And the classical pain radiating to the left arm and that. And yes. I think that’s fast to identify. (P1)

As stated above, patients with AMI can frequently be assessed by participants from a distance based on body posture and facial expressions. Nevertheless, classic AMI symptoms are not always clearly expressed, introducing uncertainty about the patient’s severity of illness and morbidity. Comprehending the patient’s suffering is an ongoing challenge. However, the more clearly the symptoms present, the easier they are for the participants to understand. When patients self-identified their situation, participants perceived a clearer expectation from the patient regarding care measures, whereas with ambiguous symptoms, there was an increased responsibility placed on participants to interpret the patient’s condition.

Participants noted that patients frequently presented with chest discomfort rather than pain. Describing this discomfort precisely proved challenging for these patients. Thus, the importance of being knowledgeable about less classical symptoms was stressed by the participants, considering that AMI presentation can vary. A patient may report chest pain but otherwise appear unaffected, occasionally misleading participants. One participant remarked:

There are many different expressions why you have to be very open for what it could be, what condition the symptom is referring to (P12)

#### Increased suffering creates a need for support and safety for patient and their relatives

This subcategory highlights the participants’ shared perspective on the significance of involving patients in their care, a task that proved challenging in instances in which communication barriers and pain hindered effective interaction. Consequently, supplementing verbal information with physical proximity was deemed necessary to enhance the patients’ sense of security. For instance, one participant sought to establish a secure environment by placing patients’ relatives alongside them in the ambulance, both for their role as medical history resources and their emotional support. Furthermore, the participants tried to reassure relatives by informing them about examination findings and what was going to happen next.

During interactions with patients, it is important to perceive each individual as a whole, recognising the uniqueness of each patient and situation. Additionally, some participants underlined the significance of taking all patients seriously, even in the absence of suspicion of an AMI. Failing to engage a patient resulted in increased difficulty in identifying the condition. Compliant patients made eliciting information regarding their symptoms and medical issues easier and more manageable. Maintaining a calm demeanour and possessing sufficient knowledge to address inquiries were considered vital. Inadequate knowledge or patient questioning could induce insecurity in the participants themselves, according to their responses.

#### Communication instils feelings of safety in stressful situations

Trust and communication emerged as essential components of nursing. Participants believed that trust was cultivated by transparently conveying suspicions to patients, irrespective of the presence of AMI suspicion:

Not to hide things from the patient, I think it is common that you don’t really want to tell the patient what you see, and it is a bit scary to tell a patient that you suspect an ongoing AMI (P4)

However, some participants noted that not all patients welcomed unfiltered information about their suspicions. Additionally, it was sometimes deemed sufficient to report on the measures taken. One participant stressed the importance of not presenting oneself as a mere information source but adapting information to the individual patient for optimal engagement. Nevertheless, participants deemed it essential to apprise the patient of forthcoming actions, as they perceived that such information was occasionally lacking on hospital arrival. Ultimately, providing information was seen as a means to alleviate suffering and instil feelings of safety, with a lack of information potentially exacerbating the patient’s distress. As one participant expressed it:

I think it’s exciting because you can prepare the patient as much as possible during the journey in, so that the patient still feels reasonably safe in this strange situation they are faced with. They are basically dragged from their home, rushed to a hospital, and then all of a sudden there are only a lot of hands around them who do a lot of things and they have a hard time influencing anything (P8)

### The time-critical patient requires knowledge and correct management

This generic category underlines a profound sense of responsibility for patient care coupled with a fear of committing errors. PENs must remain prepared for potential deterioration in the patient’s condition and may experience isolation when managing critical situations with only one colleague. In scenarios involving time-sensitive patients, prioritisation leaned towards medical interventions and immediate transportation. Education is perceived as a means to enhance both patient management and safety. Nevertheless, a lack of feedback leaves participants unaware of potential mistakes. This generic category is delineated by the subcategories: ‘Mental preparedness creates more confidence in caring’, ‘Medical measures are prioritised ahead of nursing interventions in time-critical situations’ and ‘Optimal care requires correct assessment, management and feedback’.

#### Mental preparedness creates more confidence in caring

Fear of errors and a sense of responsibility necessitated preparedness. Participants expressed apprehension not only about making mistakes but also about potential patient deterioration while they were expediting care. The fear did not stem from a lack of knowledge but from a perceived inadequacy. The experience of working alone with only one colleague intensified feelings of isolation:

It is stressful, I am not worried about what to do but more worried about whether what I do lasts all the way (P6)

Extended response times allowed for preparation alongside a colleague. Inadequate information in dispatch texts could lead to incorrect equipment or delayed mindset adjustment, impacting prompt care. Experience and knowledge bolstered confidence in managing deteriorating patients during transport and promoted reliance on others. They also reduced stress and facilitated quicker identification of time-critical cases.

#### Time-critical patients required prioritising of care measurements

Participants reported that medical measures were prioritised when encountering time-critical patients. In situations where the patient was deemed time-sensitive, the patient’s care took precedence over communication with family members and information was conveyed briefly. However, on several occasions, participants attempted to address the informational needs of relatives at a later stage, either on arrival at the hospital or by delegating this task to a colleague en route. One participant stated:

We don’t really have time for these things. So, you just must do your best but perhaps just be honest and say, we understand you’re worried, but we don’t have time to explain this now, we must go. Cause sometimes there is no time for the relatives, that’s the way it is. Then you just must prioritize to save the patient (P11)

Finally, participants emphasised that patient treatment and urgent transport to the hospital were prioritised over medical record-keeping or engaging in social interactions with the patient. Compensating for limited caring was deemed feasible at a later stage, either in the ambulance en route or after the patient’s admission to the hospital.

#### Optimal care requires correct assessment, management and feedback

Early identification was deemed critical, as time is of the essence in cases of AMI. Participants reported that when symptoms were distinctive, identifying the condition was swifter. Moreover, they observed that rapid AMI identification expedited the entire process, including ECG transmission and drug administration:

Yes, time is myocardial cells, it is what it is about. // myocardial cells dies and ischemia spreads, so time is a very important factor. So, it is good to catch them as soon as possible. It is very valuable (P7)

Although participants acknowledged the medical guidelines’ directive to transmit an ECG within 10 min, they did not prioritise it. Conversely, some participants did not perceive this as problematic, since ECG transmission could be accomplished from the ambulance en route. Additionally, participants had to prioritise interventions, not only based on the patient’s condition but also on transport time. During extended transports, participants experienced less stress as they had time to perform all procedures mandated by medical guidelines. Nonetheless, participants highlighted the value of having sufficient time to assess pain relief. One participant described their experience:

As there’s obviously one thousand things that should happen at the same time, you should speak to the doctor, get prescriptions, get the medicine into the patient. But after that’s done and you’re on the way to the hospital, there’s a lot of time for talking, explain what’s happening and above all what’s going to happen when getting to the hospital (P8)

Participants identified one component of their care responsibilities as treating symptoms and transporting the patient promptly and securely to the hospital for further treatment. Furthermore, several participants emphasised the significance of knowledge and experience in making informed choices between interventions based on the patient’s condition, which may differ depending on the patient’s unique circumstances.

Education was considered essential for enhancing knowledge, confidence and patient safety. However, numerous participants felt there was a lack of feedback regarding care provided and patient outcomes. Feedback on care provided was considered as important for the participants’ professional development. Several participants stated that they were unaware of any errors, since they did not receive any feedback on their patient management.

### Frustration when the STEMI pathway was not activated as intended

This generic category highlights the importance of effective communication and trust between healthcare providers. Communication and trust are paramount in optimising urgent patient care. Prompt activation of the STEMI pathway was also recognised as vital for delivering optimal care. However, when the STEMI pathway malfunctioned, it resulted in frustration. This generic category is delineated by the subcategories: ‘Interplay between caregivers is important for a well-functioning STEMI pathway’ and ‘The STEMI pathway saves time’.

#### Interplay between caregivers is important for a well-functioning STEMI pathway

Effective communication between participants and hospital staff was considered paramount for optimal patient management and STEMI logistics. However, participants consistently encountered situations where patients deemed to require activation of the STEMI pathway to undergo immediate percutaneous coronary intervention (PCI) were mistakenly directed to the ED instead:

You know what you have and what you want to do, but you don't receive a green signal and are directed to the emergency department, which is quite far from the cath lab. So, you had no choice but to leave the patient in the emergency department and inform them that you are coming in with a STEMI. (P4)

This issue was attributed to communication breakdowns, where cardiologists in contact with participants failed to give adequate consideration to the patient’s history or disregarded the participants’ assessments. This lack of communication caused significant frustration, resulting in participants feeling left alone with potentially time-critical patients. Moreover, the reliance on cardiologists’ assessments added to their stress levels.

A few participants suggested that enhancing their ECG interpretation skills would foster greater trust in their assessments. This became evident when the ECG transmission system failed, revealing the low level of trust in the participants’ interpretations. As a result, patients were often diverted to the ED for reassessment instead of being directly referred to the STEMI pathway for treatment.

#### The STEMI pathway saves time

The participants considered the medical guidelines a support when initiating the STEMI pathway. One participant stated:

It usually becomes obvious. We get to a patient with chest pain, register the ECG and send it, we get to know it’s priority 1 and directly to PCI. (P10)

In most cases, the participants experienced that the STEMI pathway functioning as intended. However, there were instances when the process did not operate optimally for the patient, leading to frustration and additional stress. Another participant recounted feeling frustrated and stressed in situations when an ECG could not be transmitted, and no one took responsibility for the issue; instead, the patient was referred to another hospital. This realisation that the patient’s access to prompt treatment would be delayed further exacerbated the participants’ stress and frustration. Another informant mentioned the frustration and stress that arose when the backup routines outlined in the guidelines failed. When ECG transmission was hampered due to technical errors, it became apparent that patient care would be delayed. The informants also noted that when patients lacked objective signs, such as ECG evidence of AMI, the pace of care slowed down and they were referred to the ED instead, where they had to wait with other patients for further care.

### Responsibility of care requires swift management and knowledge about the fragile life

The main category revealed that responsibility of care requires swift management and knowledge about the fragility of life. Mental preparedness was decisive for the rapid identification of the patient’s condition and for prioritising appropriate interventions. Furthermore, the importance of creating a sense of security and involvement for both patients and their relatives was seen as important. Participants achieved this by adapting their communication and level of closeness to the specific needs of each individual. Balancing patient involvement with the need for rapid medical measures posed a challenge. While they strove to keep patients informed, the urgency of treating time-critical patients with suspected STEMI often took precedence. Time was seen as central as time saves life. However, participants explained the reasons behind certain procedures to maintain patient engagement. The significance of acquiring comprehensive skills, knowledge, and experience to manage these high-risk patients effectively is highlighted. Effectively initiating the STEMI pathway involved promptly transmitting the ECG to expedite patient transport and treatment. In situations where this process did not function seamlessly, participants advocated increased trust in their knowledge and expertise.

## Discussion

The study’s aim has been to illuminate PENs’ experiences of caring for patients with suspected AMI. The findings demonstrate that patient symptoms play an essential role in prehospital emergency care assessments. Participants in this study reported operating with the understanding that patients with a potential AMI require urgent care due to its time-sensitive nature. In cases with typical symptoms, patient identification can be relatively straightforward, while atypical symptoms necessitate a more open-minded approach to determine the underlying cause. Atypical symptoms significantly complicate the assessment process, demanding a higher level of open-mindedness and knowledge of AMI from PENs to make accurate assessments and early identification of patients with AMI. This experience is also observed among nurses in cardiac care units, where it is described that patients with more ambiguous or subtle symptoms complicate the caregiving process.^15^ Studies show that atypical symptoms of AMI, particularly dyspnoea, predominantly occur among elderly patients. The absence of typical chest pain prolongs the time to hospital admission, ECG and PCI. These patients with AMI without typical chest pain suffer an increased risk of mortality, with longer hospital stays.^5 16^

Furthermore, the study’s findings indicate that it is easier to be mentally prepared for patient encounters when the information provided by the dispatch centre aligns with patient presentation on site. This allows PENs to prepare for and anticipate the patient’s condition and develop an action plan. This is supported by a previous study that found that the combination of dispatch information and experience enables ambulance personnel to prepare themselves adequately.^17^ Participants also feel a strong sense of responsibility for patient care and a need to be mentally prepared for potential patient deterioration. A fear of not being able to manage rapid patient deterioration was expressed. Participants also described needing to be able to prioritise correct actions while simultaneously remaining prepared to act swiftly in case of patient deterioration. Subsequent transitioning to a more empathetic role was described as essential. These three concerns have also been echoed in earlier research: first the need to prioritise correct actions; second readiness to act if patient deterioration occurred; and third the subsequent assumption of an empathetic role towards the patient and relatives.^17^ However, the need to be mentally prepared is not unique to caring for patients with AMI; similar observations are also seen in studies of caring for other time-critical conditions.^18 19^

Caring for patients with AMI in the EMS is complex. Limiting patient suffering, enhancing patients’ sense of safety and preparing them for further management at the hospital were perceived as crucial aspects of care. Furthermore, during the acute phase when time is critical, participants reported prioritising medical interventions over nursing care and communication, acknowledging that these aspects must temporarily take a backseat. These findings are supported by previous research that emphasises the importance of tailoring communication and care to the specific needs of each patient by adopting an individualised approach.^20 21^ This dual nature of the PENs’ role, in which PENs constantly prioritise between nursing and medical care, has also been observed in other studies.^8^ Notably, patients often experience anxiety before reaching the hospital and place their trust in the PENs and their expertise.^21^

Similar experiences are also observed intrahospital among both nurses and physicians, where medical care is perceived to need prioritisation over patient communication during the acute phase.^22 23^ Furthermore, patients tend to assume a passive role during the acute phase of AMI and that verbal communication between patients and healthcare professionals is often limited.^23^ A Swedish study involving both patients with AMI and healthcare professionals (nurses and physicians) found that patient involvement may not be as appropriate during the acute situation, but that providing information remains essential.^24^ Additionally, EMS nursing interventions, such as providing information, should be initiated parallel with medical measures.^21^ While either medical or nursing interventions may occasionally take precedence, both aspects should be integrated to ensure high-quality care.^25^ Additionally, it is known that patients often prefer less information during time-critical situations, while their need for information increases during less urgent phases. Despite this, communication has been shown to improve patient experience.^26 27^ Finally, the caring aspect of nursing encompasses treating patients as unique individuals, and this element must be incorporated into care, prioritising patient needs even though nursing may not be explicitly prioritised in guidelines or EMS care development.^8^

Minimising the time between patient arrival and ECG registration was not consistently prioritised by participants in this study. Instead, ECGs were often recorded in the ambulance rather than bringing the equipment to the patient’s home bedside. This practice is concerning, as studies have shown that earlier ECG registration can significantly reduce the time to treatment for patients with STEMI.^28 29^ Moreover, increased survival has been observed in patients for whom ECGs were registered and transmitted promptly.^29^,^31^ Previous research has also highlighted the lack of adherence to guidelines for ECG registration and transmission, with only 16% of patients meeting the target of transmitting the ECG within ten minutes of EMS arrival.^32^ Notably, adherence to guidelines for ECG registration is above 90% among patients with chest discomfort as the chief complaint.^33^ These findings underline the importance of continuing education and reinforcement of guidelines to ensure prompt ECG registration and transmission for all patients with suspected STEMI.

The study also reveals instances of frustration and lack of trust between different healthcare professions during ECG interpretation and transmission processes. When ECG transmission was hindered, participants felt their ECG interpretation skills were questioned. To address this issue, participants advocated enhanced training in ECG interpretation for PENs. However, contradictory findings from another study suggest that previous working experience in a coronary care unit is the primary factor associated with improved STEMI identification rates among nurses, while prehospital experience or education for PENs do not significantly enhance ECG interpretation skills. Additionally, this study also found that the overall knowledge of ECG interpretation among nurses working in prehospital care is relatively low.^34^ However, in contrast, the Ottawa model suggests that advanced paramedics certified in ECG interpretation can accurately identify STEMI and independently triage patients directly to the STEMI pathway with outcomes comparable to those achieved by physicians.^35^ This model highlights the potential for enhanced STEMI recognition and treatment through advanced paramedic training in ECG interpretation.

The absence of a systematic clinical performance feedback mechanism in today’s EMS hinders learning and may reduce improvement in patient outcomes. The lack of shared medical records between EMS and receiving hospitals leads to ad hoc feedback and discouragement of feedback, impeding PENs’ ability to gain valuable insights into the accuracy of their initial assessments and to refine their skills in identifying patients presenting with AMI. Furthermore, the lack of feedback is not a unique finding of this study; it has also been reported in numerous previous studies, both prehospital and intrahospital.^1836^,^38^ The transformation of the EMS from being primarily a transport-orientated service to one that provides comprehensive assessments, advanced treatments and referrals to alternative levels of care has further amplified the importance of feedback. This evolving role has placed greater decision-making responsibility on PENs, making feedback even more critical for supporting their professional development and ensuring optimal patient outcomes. Research has highlighted the negative impact of inadequate feedback on patient safety.^38 39^ While research in this area remains limited, existing evidence suggests that feedback can significantly improve adherence to guidelines and enhance clinical decision-making.^40^ Establishing a system that facilitates feedback exchange between prehospital and hospital data is crucial for promoting continuous learning and optimising patient care. Such a system would allow for prompt and comprehensive feedback on patient assessments and interventions, enabling PENs to refine their skills continuously, thus improving patient care and contributing to the overall advancement of prehospital emergency care.

### Strengths and limitations

This study expands on existing research by providing a more comprehensive understanding of the complexities of prehospital care, particularly the challenges faced in caring for patients with AMI, by incorporating the perspectives of PENs. A diverse sample was ensured by recruiting participants from different age groups, genders and working experience levels from two distinct regions. A limitation inherent to the study is that interviews were conducted solely with PENs, despite not all ambulances being staffed with these professionals. The findings may have differed if other RNs had also participated. However, the PEN has the ultimate responsibility for decisions made by the EMS team.

The study’s strength lies in the composition of the research team, which brought together expertise in both science and nursing, enabling a deeper and more nuanced interpretation of the data. Throughout the process, the utmost importance was placed on staying true to the participants’ experiences and maintaining close alignment with the data. The iterative process among authors, the back-and-forth process between the parts and the whole, further confirmed the trustworthiness of the findings. To support the credibility and transferability of the results, the findings are presented in rich descriptions supported by citations.

The recruitment of participants through voluntary responses to advertisements may have introduced an uncontrolled selection bias that could have been avoided through random sampling. The interview durations varied, but all contributed significantly valuable information. The 15 min interview remained concise and compatible with the other interviews. Another limitation could be that there were only 12 participants. However, of ultimate significance is the content and its enriching nature.

The study’s data was collected prior to the COVID-19 pandemic. While changes in patients’ care-seeking behaviour have been documented,^41^ no studies have explored potential alterations in PENs’ experiences from the pandemic’s perspective. Further research is needed to assess how the pandemic may have influenced PENs’ experiences of caring for patients with AMI.

## Conclusions

In summary, PENs grapple with immense responsibility, emphasising the need for swift and knowledgeable management in caring for patients with suspected AMI. Balancing patient involvement and urgent medical measures remains a challenge, highlighting the critical role of mental preparedness and comprehensive education. The study underlines the importance of effective communication and trust between healthcare providers for optimal patient care, especially in time-sensitive scenarios. Frustration related to STEMI pathway malfunctions reveal the necessity for streamlined protocols and enhanced trust in nurses’ expertise. Establishing feedback mechanisms for prehospital staff is imperative for learning about this patient category and for advancing prehospital emergency care.

Further investigations are warranted to assess adherence to medical guidelines. Also, a comprehensive evaluation of the healthcare chain from dispatch to the final diagnosis of AMI is essential. Comparative analyses of IT systems, communication and feedback across different countries can provide insights into prehospital AMI understanding. Our findings suggest that PENs emphasise early identification of AMI for faster and more effective care. Consequently, exploration of supplementary tools for early AMI identification is recommended for future research.

## supplementary material

10.1136/bmjopen-2024-088754 online supplemental file 1

## Data Availability

No data are available.
