# Supplementary material for: Prehospital emergency nurses’ experiences of caring for patients with suspected acute myocardial infarction: an interview study
Source: BMJ Open. 2024 Sep 10;14(9):e088754. doi: 10.1136/bmjopen-2024-088754 (PMC11409269; doi:10.1136/bmjopen-2024-088754)
Supplement: online supplemental file 1 [file bmjopen-14-9-s001.pdf]

ATTACHMENT 1. INTERVIEW GUIDE

Interview Questions:

Can you tell me about your experience of caring for patients with suspected myocardial infarction?

Follow-up questions:

Can you elaborate on (Use the participant's words)?

You mentioned (Use the participant's words) earlier...

What does (Use the participant's words) involve?

Can you describe a situation where you were involved in caring for a suspected myocardial infarction?

How did you feel about it?

What are your thoughts on this?

What emotions does this evoke in you? Can you tell me more about it?

How do you think the patient experienced your care?

For clarification:

Have I understood correctly that...?

What do you mean by (Use the participant's words)?
