# Supplementary material for: Prehospital emergency nurses’ experiences of caring for patients with suspected acute myocardial infarction: an interview study
Source: BMJ Open. 2024 Sep 10;14(9):e088754. doi: 10.1136/bmjopen-2024-088754 (PMC11409269; doi:10.1136/bmjopen-2024-088754)
Supplement: Supplementary file 2 [file bmjopen-2024-088754.reviewer_comments.pdf]

PEER REVIEW HISTORY

BMJ Open publishes all reviews undertaken for accepted manuscripts. Reviewers are asked to complete a checklist review form and are provided with free text boxes to elaborate on their assessment. These free text comments are reproduced below.

ARTICLE DETAILS

**TITLE (PROVISIONAL)**	Prehospital emergency nurses experiences of caring for patients with suspected acute myocardial infarction: an interview study
**AUTHORS**	Bjöhle, Sebastian; Vicente, Veronica; Eriksson, Caroline; Bohm, K; Dodd, Maja; Wahlin, Rebecka; Lederman, Jakob

VERSION 1 - REVIEW

**REVIEWER NAME**	Ferry, Amy
**REVIEWER AFFILIATION**	University of Edinburgh, Cardiovascular Science
**REVIEWER CONFLICT OF INTEREST**	I have no competing interests
**DATE REVIEW RETURNED**	05-Jun-2024

**GENERAL COMMENTS**	1) The manuscript describes the experience of pre-hospital emergency nurses in caring for patients with suspected acute myocardial infarction. I found the title long winded. It could be more succinctly describe as:<br>Pre-hospital Emergency Nurse experience of caring for patients with suspected acute myocardial infraction: an interview study.<br>2) Methods (page 6, line 24,35)<br>This section describes the use of the COREQ statement but not all sections on this statement are referred to 9e.g. the authors have stated N/A to most of section 1). Critically it does not state who the interviewers (SB and CE) were. Were they PEN's, social scientists, other professionals within the EMS?<br><br>I would also be interested to know how long it took to recruit 12 participants. Were all recruited in a one month period? How many potential participants came forward? Was any snowball sampling used?<br><br>Could the authors supply the interview schedule?<br><br>3) Results (page 8 line 18)<br>Could figure 1 be supplied as a table as it was difficult to get an overall sense of the categories and sub categories. I appreciate it maybe imposed by the journal style to have figures appearing at the end. If this information was supplied as a table it would provide the reader with more clarity of how the categories fit together.<br><br>4) Discussion<br>I would be interested to learn how the findings relate to the experiences of other healthcare professionals caring for suspected AMI patients in other settings (e.g. are these experiences unique to nurses?)<br>Additionally, are the experiences unique to caring for patients with AMI or patients with other time critical health conditions? It would be

	interesting to situate the findings in this body of literature if any is existing.

**REVIEWER NAME**	Turnbull, Joanne
**REVIEWER AFFILIATION**	University of Southampton, School of Health Sciences
**REVIEWER CONFLICT OF INTEREST**	None
**DATE REVIEW RETURNED**	17-Jun-2024

**GENERAL COMMENTS**	The study is a descriptive qualitative account of the experiences of 12 participants. It is clearly written in the main, rigorous enough, and offers an interesting description of findings. It lacks depth / interpretation / explanation of findings - but the paper 'does what it says' - it is a description of experiences based on a content analysis. It is not really clear enough what is new here - beyond the context of PENS being new in relation to AMI.<br>1. The introduction would benefit from a stronger statement as to what this research contributes - what are the (potential implications). Whilst it is interesting to 'illuminate PENS experiences' - how will this research contribute / what will it inform/improve? Is it about improving patient care? About nurse's clinical practice/needs? does it inform policy on PEN roles? From the intro I am just not quite clear 'why we need' this research?<br>2. The methods are clear in the main. I wasn't sure what categorised into 'generic' and 'main categories' meant?<br>3. How was data saturation 'confirmed'? What indicated data saturation? how was it judged?<br>4. How was 'researchers prior understanding' analysed? How was this gathered? How did it inform the analysis/research?<br>5. It is important to note that there was no direct observation or interviews with patients themselves. The result make various references to patient behaviours and feelings - however, it needs to be clearer that these are nurse perceptions of patient behaviours and feelings.<br>6. There is a large (theoretically informed) literature on many of the aspects that appear in the results - managing risk / role responsibility, trust and communication, clinical decision making and feedback - but none of this previous literature really informs the paper. A more sophisticated analysis would make this is a stronger paper.<br>7. The discussion highlights a number of aspects that support previous research. The paper would benefit from a stronger discussion of what this study adds to what we already know.

VERSION 1 – AUTHOR RESPONSE

Reviewer: 1

Dr. Amy Ferry, University of Edinburgh

Comments to the Author:

1) The manuscript describes the experience of pre-hospital emergency nurses in caring for patients with suspected acute myocardial infarction. I found the title long winded. It could be more succinctly describe as:

Pre-hospital Emergency Nurse experience of caring for patients with suspected acute myocardial infraction: an interview study.

Answer: Thank you for your feedback. We agree that the title was a bit too long and find your suggestion appealing. We have changed the title according to your suggestion:

Prehospital Emergency Nurses experiences of caring for patients with suspected acute myocardial infarction: an interview study.

2) Methods (page 6, line 24,35)

This section describes the use of the COREQ statement but not all sections on this statement are referred to 9e.g. the authors have stated N/A to most of section 1). Critically it does not state who the interviewers (SB and CE) were. Were they PEN's, social scientists, other professionals within the EMS?

Answer: We agree that a clearer description is needed and some statements are missing. We attach a reflexivity statement and also amended the introduction as follows:

"Both SB and CE were RNs enrolled in education to become PENs when interviews were conducted."

I would also be interested to know how long it took to recruit 12 participants. Were all recruited in a one month period? How many potential participants came forward? Was any snowball sampling used? Could the authors supply the interview schedule?

Answer: Thank you for your feedback. It took about three weeks to recruit and schedule interview appointments with the participants. All potential participants who came forward were interviewed. No snowball sampling was used in this study. The interviews were conducted between April 3 and April 19 2019 most often before or after their work shift. We agree that the execution of the interviews could have been described more clearly and have clarified the methodology as follows:

"Data collection took place face-to-face at the participants' respective workplaces between April 3 and April 19 2019 most often before or after their work shift. The interviews were conducted by the main author SB and CE under the supervision of VV and KB"

3) Results (page 8 line 18)

Could figure 1 be supplied as a table as it was difficult to get an overall sense of the categories and sub categories. I appreciate it maybe imposed by the journal style to have figures appearing at the end. If this information was supplied as a table it would provide the reader with more clarity of how the categories fit together.

Answer: It is exactly as you describe, due to the constraints imposed by the journal's style, which excludes figures at this stage. I am attaching the figure as a separate image to clarify how the categories fit together.

4) Discussion

I would be interested to learn how the findings relate to the experiences of other healthcare professionals caring for suspected AMI patients in other settings (e.g. are these experiences unique to nurses?) Additionally, are the experiences unique to caring for patients with AMI or patients with other time critical health conditions? It would be interesting to situate the findings in this body of literature if any is existing.

Answer: Thank you for your feedback. There is some research indicating that both physicians and nurses intrahospitally experience the care of AMI patients in similar ways. We have clarified the discussion as follows:

Page 17:

“Similar experiences are also observed intrahospital among both nurses and physicians, where medical care is perceived to need prioritization over patient communication during the acute phase”

These experiences are also observed in other studies, as you mentioned. We agree that this is interesting and have therefore clarified the discussion as follows:

Page 16:

“This experience is also observed among nurses in cardiac care units, where it is described that patients with more ambiguous or subtle symptoms complicate the caregiving process”

“However, the need to be mentally prepared is not unique to caring for patients with AMI; similar observations are also seen in studies of caring for other time-critical conditions”

Reviewer: 2

Dr. Joanne Turnbull, University of Southampton

Comments to the Author:

The study is a descriptive qualitative account of the experiences of 12 participants. It is clearly written in the main, rigorous enough, and offers an interesting description of findings. It lacks depth / interpretation / explanation of findings - but the paper 'does what it says' - it is a description of experiences based on a content analysis. It is not really clear enough what is new here - beyond the context of PENS being new in relation to AMI.

1. The introduction would benefit from a stronger statement as to what this research contributes - what are the (potential implications). Whilst it is interesting to 'illuminate PENS experiences' - how will this research contribute / what will it inform/improve? Is it about improving patient care? About nurse's clinical practice/needs? does it inform policy on PEN roles? From the intro I am just not quite clear 'why we need' this research?

Answer: The significance is important, and we agree that more explanation is needed. We amended the introduction as follows:

Page 6: “This study is therefore significant as it complements existing research and offer new insights into the intricate nature of caring for patients with suspected AMI. By illuminating the experiences of PENs in the EMS, we hope to improve and highlight the understanding of caring for these patients. The results of this study could also explain findings from previous and future quantitative studies.”

2. The methods are clear in the main. I wasn't sure what categorised into 'generic' and 'main categories' meant?

Answer: In the context of Elo and Kyngäs' approach to qualitative research 'generic categories' are an intermediate level of abstraction that groups subcategories together, while 'main categories' are the

most abstract and comprehensive categories that encompass all the generic categories under a unified theme or concept. I am attaching Figure 1 for a more visual overview.

3. How was data saturation 'confirmed'? What indicated data saturation? how was it judged?

Answer: Saturation was perceived to be achieved in consultation with the research team. This conclusion was reached after new interviews did not result in new codes beyond those already existing. At this stage, two additional interviews were conducted to ensure this.

4. How was 'researchers prior understanding' analysed? How was this gathered? How did it inform the analysis/research?

Answer: Throughout the research process, we continuously examined and questioned our prior understanding and judgments during team meetings and discussions to enhance reflexivity and ensure the trustworthiness of data collection and analysis. This was achieved through research group discussions that balanced subject knowledge with openness. To minimize the introduction of bias, preconceptions were critically discussed among the researchers. Additionally, subsequent transcribed interviews were independently coded by SB and CE, and the coding structure was audited by the senior supervisor VV.

5. It is important to note that there was no direct observation or interviews with patients themselves. The result make various references to patient behaviours and feelings - however, it needs to be clearer that these are nurse perceptions of patient behaviours and feelings.

Answer: Thank you for your feedback. This is indeed of utmost importance. I have reviewed the results and discussion sections and clarified areas where misunderstandings might have occurred.

6. There is a large (theoretically informed) literature on many of the aspects that appear in the results - managing risk / role responsibility, trust and communication, clinical decision making and feedback - but none of this previous literature really informs the paper. A more sophisticated analysis would make this is a stronger paper. The discussion highlights a number of aspects that support previous research. The paper would benefit from a stronger discussion of what this study adds to what we already know.

Answer: Thank you for your feedback. I have clarified in the discussion that some of the findings in this study are not unique to either the profession or the condition. We acknowledge the existence of a substantial body of theoretically informed literature on managing risk, role responsibility, trust and communication, clinical decision making, and feedback. However, our study adds a nuanced understanding of these aspects specifically within the context of prehospital emergency nursing and AMI patient care.

To strengthen our paper, we have now integrated existing literature into the discussion. We believe that our study contributes to the broader knowledge by highlighting the challenges and experiences of prehospital emergency nurses and caring for time critical patients.

We believe these revisions have strengthened the manuscript and have addressed the reviewers' concerns. We hope the revised version meets your expectations and look forward to your favorable consideration for publication.

Thank you again for your constructive feedback and support.

Sincerely,

Sebastian Bjöhle

Doctoral Student at the Department of Clinical Research and Education, Karolinska Institutet

VERSION 2 – REVIEW

**REVIEWER NAME**	Turnbull, Joanne
**REVIEWER AFFILIATION**	University of Southampton, School of Health Sciences
**REVIEWER CONFLICT OF INTEREST**	None
**DATE REVIEW RETURNED**	16-Aug-2024

**GENERAL COMMENTS**	Whilst the changes to the paper are relatively minimal, they have improved the clarity of the paper, and have added in some further points for discussion. The changes have addressed the points raised in the first review.
